# Genome-wide association study of seasonal affective disorder

**DOI:** 10.1038/s41398-018-0246-z

**Published:** 2018-09-14

**Authors:** Kwo Wei David Ho, Shizhong Han, Jakob V. Nielsen, Dubravka Jancic, Benjamin Hing, Jess Fiedorowicz, Myrna M. Weissman, Douglas F. Levinson, James B. Potash

**Affiliations:** 1 Department of Neurology, University of Florida, Gainesville, FL USA; 2 Department of Psychiatry and Behavioral Sciences, Johns Hopkins School of Medicine, Baltimore, MD USA; 3 Department of Neurobiology Research, Institute of Molecular Medicine, University of Southern Denmark, Odense C, Denmark; 4 Department of Psychiatry, University of Iowa, Iowa City, IA USA; 5 Department of Psychiatry, College of Physicians and Surgeons, Columbia University, New York, NY USA; 6 The New York State Psychiatric Institute, New York, NY USA; 7 Department of Psychiatry, Stanford University School of Medicine, Palo Alto, CA USA

## Abstract

Family and twin studies have shown a genetic component to seasonal affective disorder (SAD). A number of candidate gene studies have examined the role of variations within biologically relevant genes in SAD susceptibility, but few genome-wide association studies (GWAS) have been performed to date. The authors aimed to identify genetic risk variants for SAD through GWAS. The authors performed a GWAS for SAD in 1380 cases and 2937 controls of European-American (EA) origin, selected from samples for GWAS of major depressive disorder and of bipolar disorder. Further bioinformatic analyses were conducted to examine additional genomic and biological evidence associated with the top GWAS signals. No susceptibility loci for SAD were identified at a genome-wide significant level. The strongest association was at an intronic variant (rs139459337) within *ZBTB20* (odds ratio (OR) = 1.63, *p* = 8.4 × 10^−7^), which encodes a transcriptional repressor that has roles in neurogenesis and in adult brain. Expression quantitative trait loci (eQTL) analysis showed that the risk allele “T” of rs139459337 is associated with reduced mRNA expression of *ZBTB20* in human temporal cortex (*p* = 0.028). *Zbtb20* is required for normal murine circadian rhythm and for entrainment to a shortened day. Of the 330 human orthologs of murine genes directly repressed by Zbtb20, there were 32 associated with SAD in our sample (at *p* < 0.05), representing a significant enrichment of ZBTB20 targets among our SAD genetic association signals (fold = 1.93, *p* = 0.001). *ZBTB20* is a candidate susceptibility gene for SAD, based on a convergence of genetic, genomic, and biological evidence. Further studies are necessary to confirm its role in SAD.

## Introduction

Seasonal affective disorder (SAD) is a form of mood disorder that typically occurs in late fall and winter when periods of daylight are shortest. The hypothesis that light-sensitivity is a core feature of this disorder is supported by the efficacy of bright light and dawn simulation therapies^1^. Family and twin studies of SAD have suggested a genetic component to its etiology. Studies of the relatives of SAD probands have shown higher than expected rates of the disorder in these family members, with rates in the range of 14–26%^2–4^. A twin study demonstrated that genetic effects account for at least 29% of the variance in seasonality, including mood variation^5^.

Association studies using the candidate gene approach have been applied to investigate the role of genetic variation within biologically relevant genes in SAD susceptibility. For example, variants in genes related to serotonergic transmission, such as the serotonin transporter gene *SLC6A4*^6^ and the 5-HT_2A_ receptor gene *HTR2A*, have been associated with SAD at a suggestive level of significance^7,8^. Variants in the circadian genes *ARNTL*, *PER2*, and *NPAS2* have been reported to be associated with SAD^9,10^. A variant in the melanopsin gene, *OPN4*, which encodes a retinal protein essential for registering light intensity, was nominally associated with SAD in one report^11^.

Genome-wide association studies (GWAS) have been widely used for identifying common variants associated with complex diseases. In contrast to the candidate gene approach, GWAS allows for testing genetic variants across the genome without reliance on prior knowledge about disease biology. Genetic findings from GWAS are shedding new light on psychiatric disorders, such as depression, schizophrenia, and bipolar disorder^12–14^. However, GWAS of SAD has to date been limited to one study, which did not report any promising candidate genes^15^.

In the current study, we performed a meta-analysis of GWAS for SAD in 1380 cases and 2937 controls of European-American (EA) origin, selected from samples for GWAS of major depressive disorder (MDD) and of bipolar disorder (BD). We report that *ZBTB20* is a candidate susceptibility gene for SAD, based on a convergence of genetic, genomic, and biological evidence.

## Methods

### Study samples

Case-control samples for SAD GWAS were selected from those originally ascertained and assessed for MDD or BD. Informed consent for participation in genetic studies was obtained from all subjects. Basic information for the study samples from each GWAS is summarized in Table 1. The following provides details on the samples included.

**Table 1:** Basic information for the study samples from each GWAS

Samples	Platform	SAD	Non-SAD	Controls
GAIN	Affymetrix 6.0	296	427	1034
TGEN	Affymetrix 6.0	436	665	401
TGEN2	Affymetrix 6.0	86	147	80
GENRED	Affymetrix 6.0	325	671	492
GENRED2	Illumina Omni1-Quad	237	554	930
Total	—	1380	2464	2937

*SAD* seasonal affective disorder, *non-SAD* nonseasonal affective disorder

BD subjects were selected from the National Institute of Mental Health (NIMH) Bipolar Genetics Study (BiGS). The genotyping focused on European ancestry subjects with Bipolar I (BPI) disorder from among those collected by the NIMH Genetics Initiative for Bipolar Disorder in five waves at 11 sites across the United States, as described elsewhere in detail^16^. Only BPI cases were included in this analysis. All cases were interviewed with the Diagnostic Interview for Genetic Studies (DIGS), and these together with interviewer observations, medical records and family history data collected through the Family Interview for Genetic Study (FIGS), were used to assign diagnoses based on DSM-III or DSM-IV criteria following a best-estimate procedure. Details of the DIGS and FIGS are available at http://www.nimhgenetics.org.

MDD subjects were selected from the Genetics of Recurrent Early-Onset Depression (GenRED) study. GenRED I collected affected sibling pair families for linkage studies, while GenRED II recruited cases for association studies. Probands had MDD diagnosed with either >1 episode or a single episode lasting >3 years, onset before age 31, and a parent or a sibling with MDD and age of onset <41. Proband information was gathered through the DIGS, which together with an interviewer narrative summary, available medical records and family history data gathered through FIGS, was used to establish a diagnosis based on DSM-IV criteria. Ascertainment and assessment details for GenRED have been previously described^17^.

Healthy control samples were obtained from the NIMH Genetics Initiative repository. Some of these were selected from among those ascertained through an NIMH-supported contract mechanism between Dr. Pablo Gejman and Knowledge Networks, Inc.^18^. They were ascertained to be free of psychiatric illness based on self-report using the Composite International Diagnostic Interview—Short Form, which assesses major depression, various anxiety disorders, and alcohol and drug dependence. This was augmented with three items inquiring about lifetime diagnosis of, or treatment for, schizophrenia, schizoaffective disorder, and bipolar disorder. A total of 1515 of these healthy control European ancestry (EA) subjects were included for the BD GWAS. Another independent set of 1422 healthy EA controls were included for the MDD GWAS, which included 492 from the above data set, and 930 more from the Genomic Psychiatry Cohort or the Mayo Clinic Biobank.

### Phenotype assignment

The assignment of the seasonal or nonseasonal case status was based on identifying all subjects with seasonal depressive episodes among BD or MDD patients. We used a broad definition of seasonal depression. The assignment of seasonal depression status was based on the subject’s answer to “Do your depressions tend to begin in any particular season?” question in DIGS. All subjects with winter or fall answers were assigned seasonal case status in our study, and all of the others with answers became nonseasonal case subjects (non-SAD).

### GWAS and quality control

All BD GWAS samples were genotyped with the Affymetrix 6.0 genome-wide SNP array at the Broad Institute Center for Genotyping and Analysis. Different genotyping efforts led to a partition of BiGS data into two sets: those genotyped as a part of Genetic Association Information Network Bipolar Sample (GAIN) and those genotyped as a part of the Translational Genomic Research Institute Sample (TGEN). The GAIN set includes subjects from the first four waves of the NIMH study, and a small subset of wave five subjects. The TGEN set includes most of the wave five subjects. GenRED samples were genotyped in two waves. First wave samples were genotyped using the Affymetrix 6.0 genome-wide SNP array, and the second wave used the Illumina Omni1-Quad microarray (GenRED2).

Initial quality control (QC) for each GWAS data set removed subjects that failed set thresholds for call rates or heterozygosity. We carried out additional QC filtering for each data set with the following criteria: minor allele frequency (MAF) > 1%, missing rate per SNP < 5%, and Hardy−Weinberg equilibrium (HWE) *p* value > 10^−6^. After QC, there were 692,411 (GAIN), 701,183 (TGEN), 701,402 (TGEN2), 646,301 (GENRED), and 719,050 (GENRED2) autosomal SNPs retained for imputation. To investigate population stratification, we computed principal components (PC) for GWAS samples using EIGENSOFT^19^ based on a subset of SNPs that were in low linkage disequilibrium with one another (*r*^2^ < 0.2). We did not detect any outlier subjects from the PC analysis, which were defined as subjects whose ancestry was at least three standard deviations from the mean on one of the two largest PCs.

### Imputation

Following the best practice guidelines of IMPUTE2^20^, we imputed 1000 Genomes variants into each GWAS sample. Prephasing was first performed with SHAPEIT^21^ to infer haplotypes for samples based on autosomal SNPs with MAF > 0.01. Imputation was carried out on prephased haplotypes using IMPUTE2 against haplotype reference data from the 1000 Genomes Phase 3. After postimputation QC (SNP missing rate < 0.05, MAF > 0.01, imputation quality score > 0.5, and HWE > 10^−6^), there were 8,462,922 (GAIN), 8,458,360 (TGEN), 8,272,185 (TGEN2), 8,452,433 (GENRED), and 8,498,837 (GENRED2) autosomal variants, among which the majority of variants were imputed, with an average proportion of 92% across study samples. A common set of 8,153,767 SNPs were retained for meta-analysis.

### Association analysis

We first carried out association analysis for each autosomal SNP with SAD in each sample, and then performed meta-analysis to combine association evidence across samples. In association analysis, to account for the uncertainty of imputed genotypes, expected allelic dosages were used in a logistic regression framework as implemented by SNPTEST^22^. Sex and the first three PCs of population structure were included as covariates. We carried out meta-analysis based on effect size and standard error using METAL under a fixed-effect model^23^. We evaluated the possibility of population stratification or other systemic biases by using the quantile−quantile (QQ) plots based on *p* values of SNPs. Regional association plots were created using the online web tool LocusZoom^24^.

We computed gene-based *p* values using SimpleM^25^ to evaluate the association evidence at the gene level. SimpleM takes the smallest *p* value of SNPs within a gene while correcting for the effective number of independent SNPs through Bonferroni correction. The effective number of independent tests was estimated from the correlation matrix of SNPs using PC analysis, so that the corresponding eigenvalues explain 99.5% of the variation for the SNP data. We assigned a SNP to a gene if it was located within the gene, based on hg19 refGene annotation, or within 20 kb upstream or downstream of the gene, to capture regulatory variants.

### Expression QTL (eQTL) analysis

To determine whether the lead SNP from association analysis has an effect on regulating gene expression in specific brain regions, we queried the brain eQTL online database (http://www.braineac.org/), which contains eQTL information for ten human brain regions. Full details on the database are available elsewhere^26^.

### Enrichment analysis for ZBTB20 candidate target genes

We examined whether ZBTB20 candidate target genes were enriched for genes associated with SAD. Specifically, a two-sided Fisher’s exact test was used to determine whether there was an overrepresentation of nominally significant genes (*p* ≤ 0.05) among ZBTB20 candidate targets in comparison to the remaining genes. Candidate target genes of ZBTB20 were derived from a previous study, in which ChIP-Seq combined with microarray gene expression analysis approaches were applied to identify 346 murine genes directly repressed by Zbtb20 during hippocampal CA1 neurogenesis in vivo^27^. The 346 murine genes were mapped to 342 human orthologous ZBTB20 candidate target genes, of which 330 were assigned a gene-level *p* value for SAD.

## Results

### Association analysis

**Fig. 1:**
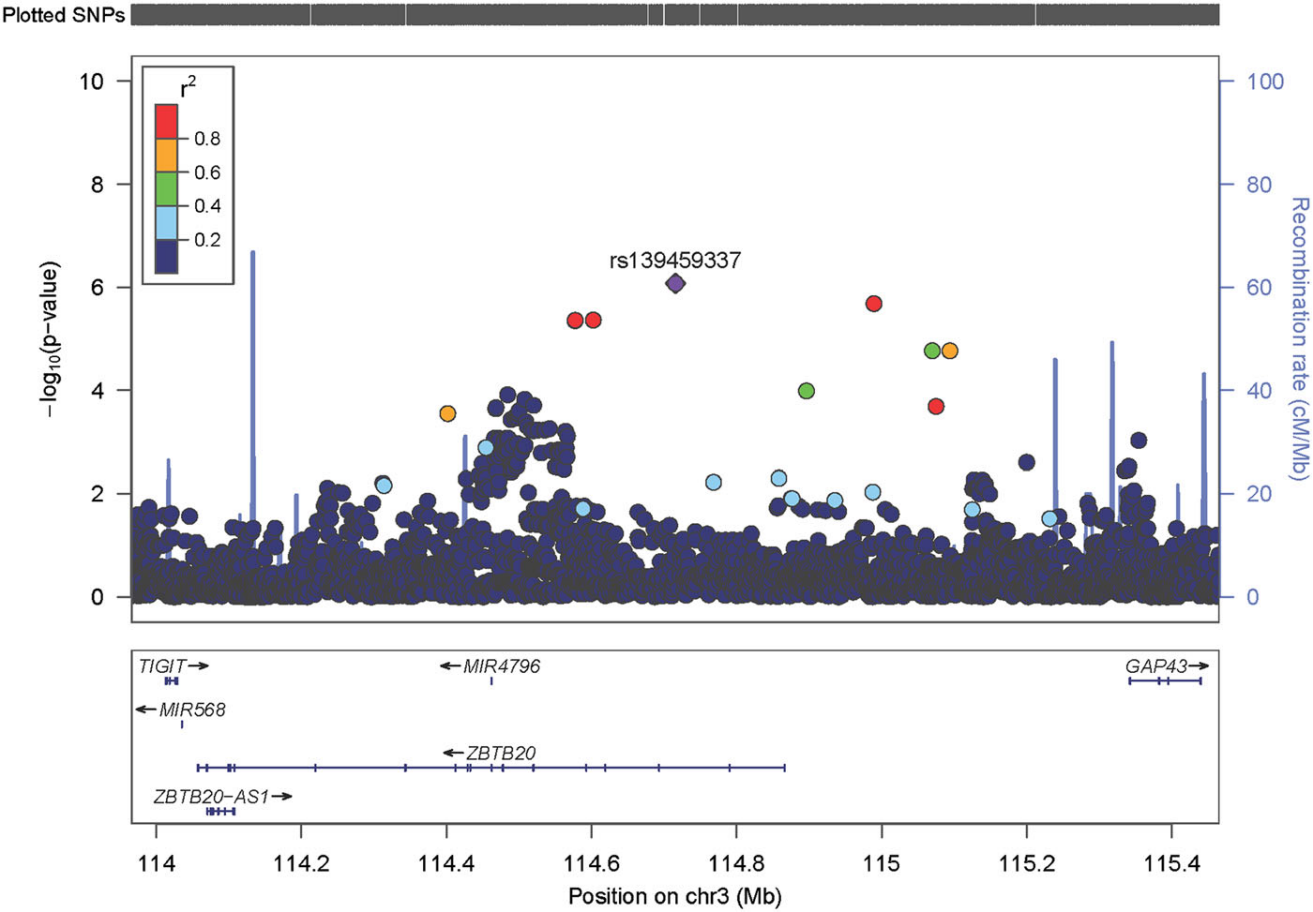
Regional association plot of SNPs in and around the top candidate gene *ZBTB20*. SNPs are plotted with their −log10 (*p* value) on the *y-*axis along with their physical position (NCBI build 36) on the *x-*axis. The SNPs are color coded according to their correlations (*r*^2^), with the most significant SNP rs139459337 shown in purple. The light blue line and right *y-*axis indicate the observed recombination rates in the HapMap CEU samples

The Manhattan plot for the meta-analysis of GWAS for SAD is illustrated in Figure S1. The QQ plot of *p* values indicated minimal evidence of population stratification or other systematic bias (lambda = 1.0, Figure S2). Overall, no single SNPs were identified at genome-wide significance (*p* < 5×10^−8^). There were 90 SNPs associated with SAD at *p* < 1×10^−5^ (Table S1). The strongest association evidence was at an intronic variant (rs139459337) within *ZBTB20* (odds ratio (OR) = 1.63, *p* = 8.4×10^−7^), which encodes a transcriptional repressor that plays a key role in neurogenesis. Figure 1 shows the regional association plot for variants within and around *ZBTB20*.

Table 2 provides details of the association analysis results for the lead SNP rs139459337. The minor allele “T” was associated with an increased risk for SAD in each sample. We found no significant association for rs139459337 with non-SAD (*p* = 0.76, Table S1), suggesting the top GWAS signal was specific to SAD. A broader case-only GWAS comparing SAD to non-SAD cases did not reveal compelling signals; however, rs139459337 differed between SAD and non-SAD with OR of 1.47, and *p* value of 5.9×10^−5^ (Table S1).

**Table 2:** Association between rs139459337 and SAD in each sample and meta-analysis

Samples	Info	Minor allele	MAF (cases)	MAF (controls)	OR	95% CI	*p*
GAIN	0.95	T	0.093	0.062	1.59	1.13–2.25	0.01
TGEN	0.94	T	0.087	0.055	1.94	1.26–2.99	0.0021
TGEN2	0.80	T	0.078	0.063	1.45	0.51–4.12	0.49
GENRED	0.93	T	0.077	0.052	1.55	0.99–2.43	0.053
GENRED2	0.96	T	0.089	0.069	1.55	1.05–2.28	0.03
Meta-analysis	—	T	0.086	0.062	1.63	1.34–1.98	8.4×10^−7^

*Info* imputation quality score from Impute2, *MAF* minor allele frequency, *OR* odds ratio, *CI* confidence interval

We also evaluated the genetic association evidence at the gene level for the SAD vs. healthy control comparison; however, no genes reached genome-wide significance (*p* < 2×10^−6^). The strongest gene-level association evidence was, again, for *ZBTB20* (*p* = 8.0×10^−5^, Table S2).

### eQTL analysis

We queried a brain eQTL database and examined whether the lead SNP rs139459337 regulates *ZBTB20* mRNA expression levels in ten brain regions. In this small sample, we observed that the risk allele “T” was associated with lower *ZBTB20* mRNA levels in nine of the ten regions, a difference that was nominally significant in the temporal cortex (*p* = 0.028) (Figure S3). The result does not survive correction for multiple testing.

### Enrichment of GWAS signals for ZBTB20 candidate target genes

**Fig. 2:**
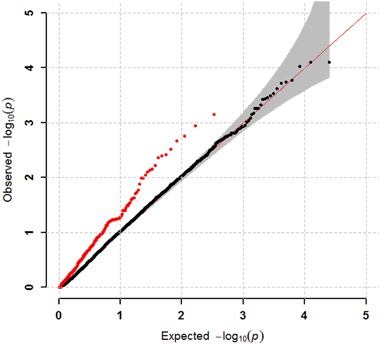
Stratified quantile−quantile (QQ) plot for ZBTB20 candidate gene targets (red) and nontarget genes from SAD GWAS (black). The gray shading indicates 95% confidence interval under the null hypothesis of no association

Because both SNP and gene-level association analyses implicated *ZBTB20* as our strongest finding, we tested whether there was an enrichment of SAD GWAS association signals for genes that are candidate targets for the ZBTB20 transcriptional repressor protein. Of the 330 human orthologues of murine genes directly repressed by Zbtb20, we found that 32 were associated with SAD (*p* < 0.05), representing a significant enrichment as compared to chance expectation (fold enrichment = 1.93, *p* = 0.001). When we examined the QQ plot of *p* values, we found an obvious deviation from the uniform expected distribution for *p* values of the ZBTB20 candidate target gene group, but not for the remaining nontarget genes (Fig. 2). Table S3 provides a list of ZBTB20 targets that showed nominally significant association with SAD (*p* < 0.05).

### Candidate genes

A number of candidate genes of biological interest have previously been tested for their association with SAD. These include genes related to serotonergic transmission, such as *SLC6A4*^6^ and *HTR2A*^7,8^; circadian genes, such as *ARNTL*, *PER2* and *NPAS2*; and *OPN4*, which encodes the photoreceptive opsin protein. Using the number of common SNPs (MAF > 0.01) that appear in the CEU samples of 1000 Genomes as a reference, we first evaluated whether our two genotyping platforms (Affymetrix 6.0 and Illumina Omni1-Quad) provided adequate coverage of SNPs in these candidate genes. Although the two genotyping arrays contained only a limited proportion of SNPs within each candidate gene (ranging from 4 to 19%), imputation greatly improved the coverage of these SNPs (89−97%, Table S4). In our current GWAS, none of these genes yielded SNPs with association *p* values < 1×10^−3^. The gene with the strongest association evidence was *NPAS2*, which included the SNP rs113837129, associated with SAD at *p* = 0.001. Gene-level analysis of *NPAS2* produced a trend toward association (*p* = 0.09). Gene-level analyses did not show any significant association evidence for other candidate genes (*p* > 0.3). Table S5 provides the SNP-level analysis results with nominal significance for these candidate genes.

## Discussion

In the current study, we conducted a GWAS for SAD in an EA sample. Although no genome-wide significant findings were identified, we report that *ZBTB20* is a strong candidate susceptibility gene for SAD based on a convergence of genetic, genomic, and biological evidence. In particular, the lead SNP rs139459337 within *ZBTB20* showed modest eQTL evidence for regulating gene expression in temporal cortex. In addition, ZBTB20 candidate targets were enriched for genes nominally associated with SAD.

Intriguingly, prior research suggests there is substantial biological plausibility for *ZBTB20* as a candidate gene for SAD. ZBTB20 is a zinc finger transcriptional repressor protein that is particularly abundant in the hippocampus^27^. The murine *Zbtb20* gene is essential for the specification of hippocampal CA1 field identity^28^. Ectopic expression of Zbtb20 proteins in transgenic mice causes behavioral abnormalities suggestive of deficiency in visual and spatial memory processing^29^. Most strikingly, a recent study reported that loss of Z*btb20* in mice impairs circadian rhythms, particularly reducing activity in the early evening period of those nocturnal animals^30^. The Z*btb20-*deficient mice also had more difficulty than control mice in entraining to a shortened day.

Haploinsufficiency of *ZBTB20* has been reported to be involved in the neurodevelopmental and neuropsychiatric disorders seen in the 3q13.31 microdeletion syndrome^31^ and in Primrose syndrome^32^. Interestingly, our eQTL analysis indicated, albeit at a suggestive level, that the risk allele of rs139459337 decreases *ZBTB20* expression in temporal cortex, which is in accordance with the haploinsufficiency mechanism of *ZBTB20* in neuropsychiatric disorders. The reduced gene expression level of the risk allele also parallels a previous study, which reported that hypermethylation in the *ZBTB20* gene was associated with MDD^33^.

Our analysis has shown significant enrichment of SAD association signals for candidate gene targets of ZBTB20. This result implies that pathways regulated by *ZBTB20* might be important in the etiology of SAD. A number of candidate gene targets of ZBTB20 are worth noting, as they have been implicated in various psychiatric disorders and behavioral phenotypes. These include *NRXN1*^34^, *NRXN3*^35^, and *SYN3*^36^. The neurexins are a family of cell adhesion molecules that act predominantly at the presynaptic terminal in neurons and play essential roles in neurotransmission and differentiation of synapses. *NRXN1* and *NRXN3* have both been strongly implicated in autism, and NRXN1 has been associated with schizophrenia^37^. There have also been several studies suggesting a relationship of these genes to mood disorders. Deletions in *NRXN1* were associated with poorer response to antidepressant medications^38^. A SNP in *NRXN3* was associated, at a genome-wide significant level, with symptom improvement during citalopram/escitalopram treatment, though this did not replicate in additional samples^39^. Both *NRXN1* and *NRXN3* have altered expression levels in postmortem brains of suicide completers^40^. Interestingly, *NRXN1* has also been suggested to play a role in circadian rhythm. Diurnal rhythms in mNRXN1 transcription were found in the superchiasmatic nucleus of mice^41^. *SYN3* is a member of the synapsin gene family encoding neuronal phosphoproteins that associate with the cytoplasmic surface of synaptic vesicles. The SYN3 protein has a role in synaptogenesis and the modulation of neurotransmitter release. *SYN3* was implicated in a study that examined DNA methylation differences in MZ twins selected based on discordance in cortisol and MRI measures related to anxiety^42^.

Our analysis of candidate genes yielded little evidence of association with SAD, though it did turn up nominally significant association in *NPAS2*. Notably, like Zbtb20, Npas2 plays a role in circadian rhythms. It is closely related in primary amino acid sequence to Clock, a transcription factor expressed in the suprachiasmatic nucleus (SCN) that regulates circadian rhythm. Npas2 was identified as an analog of Clock operating as a circadian regulator in the mammalian forebrain^43^. Later, it was shown that Npas2 can functionally substitute for Clock in the SCN in mice to regulate circadian rhythmicity^44^. In one study *Npas2*-deficient mice showed a number of subtle differences in circadian activity as compared to wild-type mice. Under normal 12-h light/12-h dark cycles followed by constant darkness conditions, *Npas2* (+/–) and *Npas2* (–/–) mice displayed rhythmic locomotor activity largely comparable to that of wild-type littermates. The exception is that *Npas2* (–/–) mice postponed or skipped a prominent break present in wild-type mice during the middle of the night^45^. By comparison, *Zbtb20* (–/–) mice, when compared with the *Zbtb20* (+/–) and control mice, displayed a dramatic loss of early evening activity along with significantly limited durations in their early morning activity^30^. In terms of rhythmic oscillation of locomotor activity, it was decreased in *Npas2* (–/–) mice compared to the wild-type and (+/–) mice. On the other hand, the intrinsic period was increased in *Zbtb20* (–/–) mice compared to the control mice.

This study should be considered in light of several limitations. First, our assessment of seasonality was retrospective. However, given that we were asking about a recurring pattern, rather than a one-time event, recall should be reasonably robust. Retrospective assessment of SAD has also been found to be reliable^45^. A second limitation lies in our definition of seasonality. We did not use assessment tools such as the Seasonal Pattern Assessment Questionnaire (SPAQ) to quantify seasonality based on the Global Seasonality Score (GSS)^46,47^. Therefore, we are not able to examine seasonality as a continuous phenotype. Finally, our sample size was small for a GWAS study, and thus our power to detect genome-wide significant associations was limited.

In conclusion, our study identifies *ZBTB20* as a candidate susceptibility gene for SAD. Further study in independent samples with the appropriate phenotype data is needed to confirm this association.

## Electronic supplementary material

Supplementary Figure Legends

Table S1

Table S2

Table S3

Table S4

Table S5

FigS1

FigS2

FigS3
