# Supplementary material for: Genome-wide association study of seasonal affective disorder
Source: Transl Psychiatry. 2018 Sep 14;8:190. doi: 10.1038/s41398-018-0246-z (PMC6138666; doi:10.1038/s41398-018-0246-z)
Supplement: Supplementary file 1 — Supplementary Figure Legends [file 41398_2018_246_MOESM1_ESM.docx]

**Supplementary Figure Legends**

**Figure S1.** Manhattan plot of the genomewide association analysis results of SAD. The *x* axis represents genomic coordinates, and the *y* axis represents the –log10 (p-value) for each SNP. The dashed horizontal blue line indicates a p-value of 1 × 10^-5^.

**Figure S2.** Quantile-quantile plot (QQ) for SNP-level association analysis results for SAD.

**Figure S3.** Association of rs139459337 with *ZBTB20* mRNA expression level in ten brain regions from BRAINEAC (http://www.braineac.org/). *SNIG* substantia nigra, *PUTM* putamen, *MEDU* the inferior olivary nucleus, *THAL* thalamus, *OCTX* occipital cortex, *HIPP* hippocampus, *FCTX* frontal cortex, *TCTX* temporal cortex, *WHMT* intralobular white matter, *CRBL* cerebellar cortex.
