# Supplementary figures and images for: Genome-wide association study of seasonal affective disorder
Source: Transl Psychiatry. 2018 Sep 14;8:190. doi: 10.1038/s41398-018-0246-z (PMC6138666; doi:10.1038/s41398-018-0246-z)

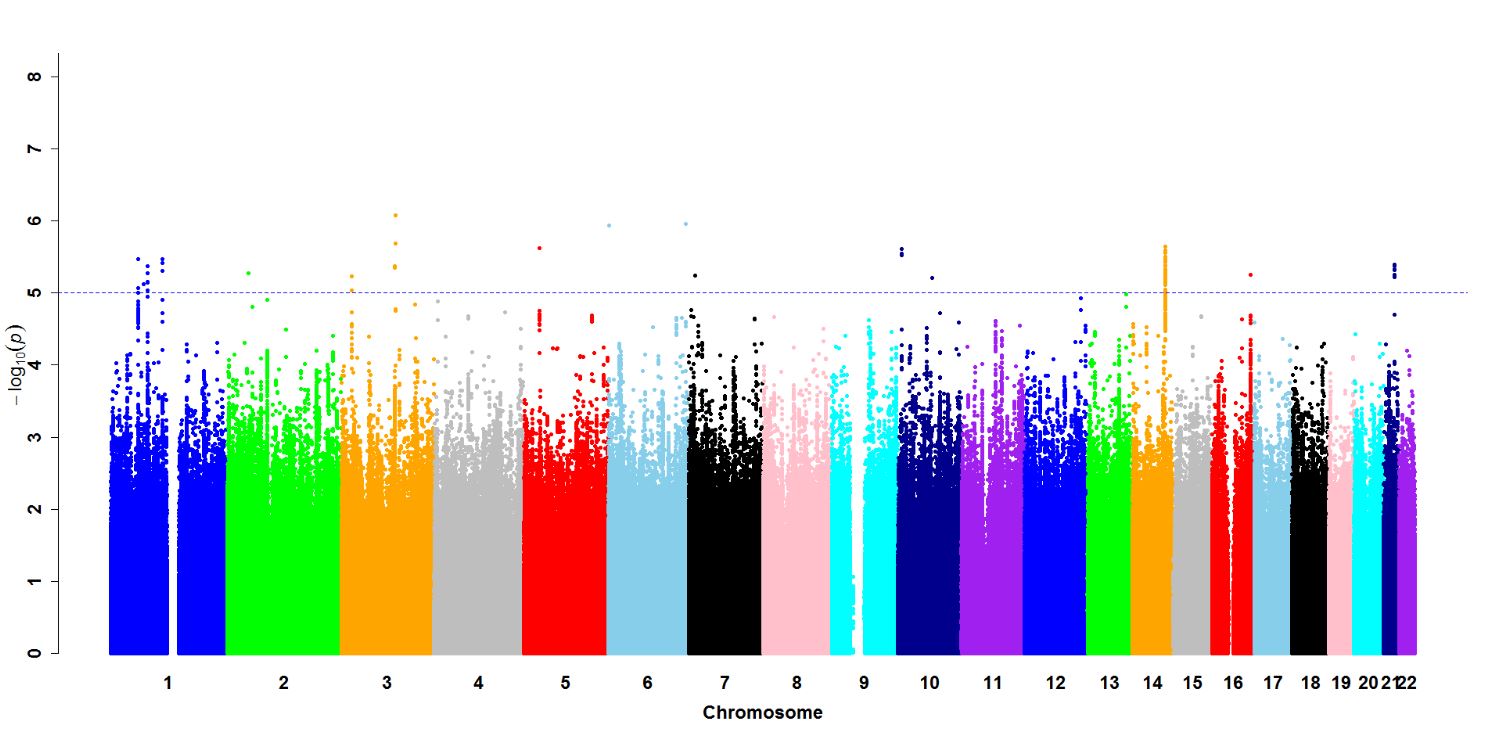

Supplement: Supplementary file 7 — FigS1 [file 41398_2018_246_MOESM7_ESM.jpg]

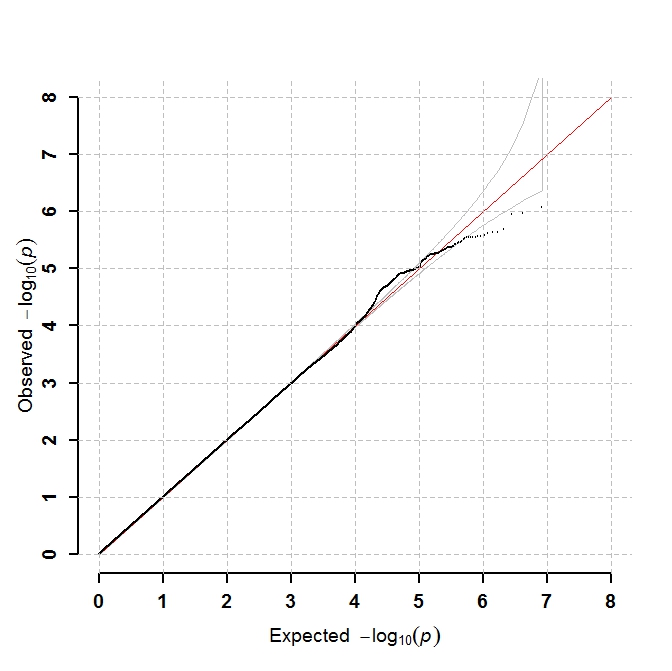

Supplement: Supplementary file 8 — FigS2 [file 41398_2018_246_MOESM8_ESM.jpg]

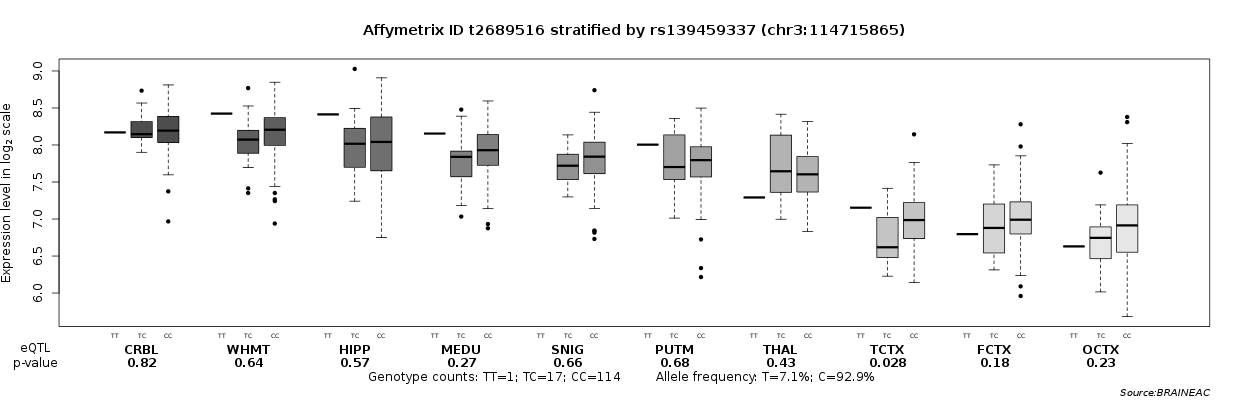

Supplement: Supplementary file 9 — FigS3 [file 41398_2018_246_MOESM9_ESM.jpg]
